# Side-by-side comparison of BH3-mimetics identifies MCL-1 as a key therapeutic target in AML

**DOI:** 10.1038/s41419-019-2156-2

**Published:** 2019-12-04

**Authors:** Larissa Ewald, Jessica Dittmann, Meike Vogler, Simone Fulda

**Affiliations:** 1 Institute for Experimental Cancer Research in Pediatrics, Goethe-University Frankfurt, Frankfurt, Germany; 2 German Cancer Consortium (DKTK), Partner Site Frankfurt, Frankfurt, Germany; 3 German Cancer Research Center (DKFZ), Heidelberg, Germany

**Keywords:** Cancer therapeutic resistance, Targeted therapies

## Abstract

Despite advances in the treatment of acute myeloid leukemia (AML), prognosis of AML patients is still dismal and better treatment options are required. B-cell Lymphoma 2 (BCL-2) homology domain 3 (BH3)-mimetics are emerging as a novel class of apoptosis-inducing agents that are currently being tested for the treatment of different hematological malignancies including AML. Particularly, the selective BCL-2 inhibitor ABT-199/Venetoclax is demonstrating clinical responses and has recently been approved in combination for the treatment of AML. Compounds targeting the related protein MCL-1 have recently entered clinical trials, highlighting the urgency to compare the different BH3-mimetics and identify the most promising antiapoptotic target in AML. We performed a side-by-side comparison of different highly selective and potent BH3-mimetics targeting BCL-2 (ABT-199), MCL-1 (S63845) or BCL-x_L_ (A1331852) in a panel of AML cell lines and primary patient cells. Gene knockdown using siRNAs was utilized to investigate the functional relevance of BCL-2 proteins. Western blotting and immunoprecipitations were used to explore the influence of BH3-mimetics on interactions between pro- and antiapoptotic BCL-2 proteins. A1331852 induced apoptosis only in selected cases, indicating that BCL-x_L_ is not a very promising therapeutic target in AML. However, S63845 displayed higher potency than ABT-199, with more cell lines and primary cells responding to S63845 than to ABT-199. MCL-1 dependency in AML cells was confirmed by siRNA-mediated knockdown of MCL-1, which was sufficient to induce apoptosis. S63845-induced cell death was accompanied by a displacement of the BH3-only protein BIM as well as BAK, resulting in BAK-dependent apoptosis. In contrast, ABT-199-induced cell death was mediated by BAX rather than BAK, indicating distinct non-redundant molecular functions of BCL-2 and MCL-1 in AML. Our study reveals that MCL-1 may be a more prevalent therapeutic target than BCL-2 in AML and identifies BIM and BAK as important mediators of S63845-induced apoptosis in AML.

## Introduction

AML is a heterogeneous myeloid disease in which normal bone marrow function is disturbed by a population of infiltrating malignant cells. Without treatment, AML is a highly aggressive disease and often fatal within weeks of diagnosis. Treatment of AML may depend on the subtype of the disease, but most often involves chemotherapy. Despite advances in the treatment of AML, the disease is only cured in 35% of patients under 65 years of age and even less in older patients, highlighting the urgent need for better treatment options^1^.

AML cells are derived from myeloid precursor cells and characterized by a failure to differentiate correctly or to undergo apoptosis. Clonal expansion of malignant cells is driven by genetic mutations that provide a survival advantage. Interestingly, some of these somatic mutations may already be present in pre-leukemic stem cells and therefore their importance as therapeutic targets remains unclear^2^. The outgrowth of a dominant subpopulation is often accompanied by changes in the apoptotic signaling network. Therefore, targeting apoptosis may represent a promising therapeutic strategy in AML that may work independently of individual mutations or patient characteristics. Intrinsic apoptosis is regulated by BCL-2 proteins which control the release of cytochrome *c* from mitochondria into the cytosol. Once released into the cytosol, cytochrome *c* facilitates the formation of the apoptosome and activation of caspase-9. The BCL-2 protein family contains the proapoptotic multidomain proteins BAX and BAK, the proapoptotic BH3-only proteins as well as antiapoptotic proteins such as BCL-2, BCL-x_L_, and MCL-1. Although BCL-2 proteins may exert multiple cellular functions, e.g. in regulating mitochondrial physiology and nuclear processes (reviewed in^3^), the main function of the antiapoptotic BCL-2 proteins in cancer cells is to prevent mitochondrial apoptosis. They bind and sequester proapoptotic BCL-2 proteins, thereby preventing the activation of BAX and/or BAK and their ability to form pores in the outer mitochondrial membrane (OMM) through which cytochrome *c* can be released into the cytosol^4^. Due to their central role in maintaining mitochondrial integrity, antiapoptotic BCL-2 proteins represent important therapeutic targets in cancer^5^, and the potential of BH3-mimetics to induce apoptosis in AML has been elucidated by multiple studies^6–11^. While early BH3-mimetics such as ABT-737^12^ or ABT-263/Navitoclax^13^ targeted multiple BCL-2 proteins, the development of ABT-199/Venetoclax demonstrated that, despite functional and structural similarities of the different antiapoptotic BCL-2 proteins, selective targeting of individual BCL-2 proteins is possible^14^.

Several BH3-mimetics are currently being tested in clinical trials for the treatment of AML^15^. ABT-199 in combination with decitabine or azacitidine has led to a complete remission in 67% of patients, highlighting the potential of BH3-mimetics in the treatment of AML^16^. These impressive results have led to accelerated Food and Drug Administration (FDA) approval for the treatment of older AML patients. Other combinations of ABT-199, e.g. with targeted agents are currently being tested in early stage clinical trials (reviewed in^8^). Besides BCL-2, also the related antiapoptotic protein MCL-1 is being discussed as a potential therapeutic target in AML^17,18^. In particular, the recently developed BH3-mimetic S63845 displayed high activity in preclinical models of AML^19^. Sensitivity to S63845 was shown to inversely correlate with the mRNA expression levels of BCL-x_L_ but not BCL-2. Currently, early clinical trials with three different MCL-1-targeting BH3-mimetics (S64315, AMG176^20^, AZD5591) have been launched (NCT02979366, NCT02675452, NCT03218683). A fourth preclinical MCL-1 inhibitor, i.e. VU661013, has recently been shown to induce apoptosis in AML and to overcome resistance to ABT-199^21^.

The importance of BCL-x_L_ as therapeutic target in AML has been less investigated. However, BCL-x_L_ has been identified as a resistance factor for both MCL-1 and BCL-2 inhibitors, highlighting its potential importance in the treatment of AML^10,19^. Selective and potent inhibitors of BCL-x_L_ have only recently been developed^22^, but their efficacy in AML has not yet been investigated in detail. In this study, we aimed to compare BCL-2, MCL-1 and BCL-x_L_ as therapeutic targets in AML and to investigate the molecular mechanisms of selective inhibitors targeting these related antiapoptotic proteins.

## Results

### A side-by-side comparison of BH3-mimetics reveals more pronounced responses to S63845 than to ABT-199 or A1331852

**Fig. 1:**
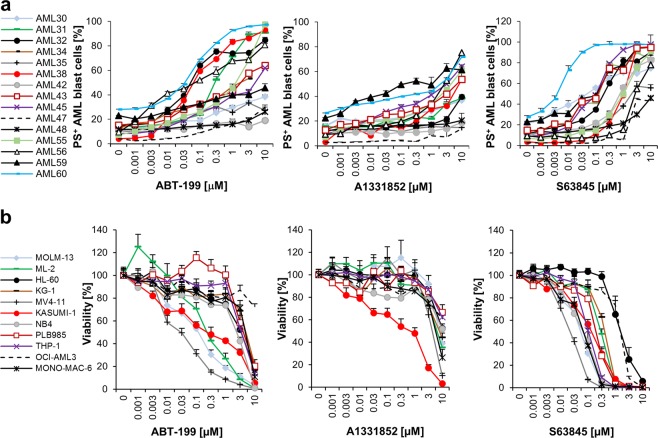
MCL-1 represents amongst the BCL-2 proteins the most promising therapeutic target in AML. **a** Primary AML samples were treated with indicated concentrations of ABT-199, A1331852 or S63845 for 20 h. Cell death was measured by FACS analysis using Annexin-V/FITC staining in combination with anti-CD45/APC staining. Mean of each experiment performed in duplicate or triplicate is shown. **b** Eleven AML cell lines were treated with indicated concentrations of ABT-199, A1331852 or S63845 for 72 h prior to analysis of viability using CellTiter-Glo. Data shown are mean and standard error of mean (*n* = 3–7).

To investigate which BCL-2 protein may be the most important therapeutic target in AML we directly compared the BCL-2 inhibitor ABT-199, the BCL-x_L_ inhibitor A1331852 and the MCL-1 inhibitor S63845 in primary blast cells isolated from AML patients (Fig. 1a). In total, 15 patient samples were treated with ABT-199 and 14 samples were treated with A1331852 or S63845. All selective BH3-mimetics were able to induce cell death in some cases at nanomolar concentrations, with ABT-199 and S63845 displaying higher efficiency than A1331852. A direct comparison of the EC_50_ values for ABT-199 and S63845 revealed significantly better responses to S63845 than to ABT-199 (Fig. S1). The response to BH3-mimetics was highly heterogeneous, with some samples responding best to ABT-199 (e.g., #32, #38 and #56), but others displaying higher sensitivity to S63845 (e.g. #42, #43, #45 and #60). Sensitivity to BH3-mimetics appeared to be independent of clinical characteristics, although sample numbers were not statistically significant (Table 1). These data demonstrate that the response to selective BH3-mimetics targeting these antiapoptotic BCL-2 proteins is not homogeneous and varies from patient to patient.

**Table 1:** Characteristic of AML primary cells and cell lines.

Primary AML	FAB	WHO classification	Age	Blasts	ABT-199	A1331852	S63845
			[Y]	[%]	EC_50_ [µM]	EC_50_ [µM]	EC_50_ [µM]
30*	M5		75	75	>10	>10	0.06
31*		AML without maturation	83	87	0.38	>10	0.1
32*			47	34	0.04	>10	0.22
34		AML with NPM1 mutation	58	91	>10	N.D.	N.D.
35		sec. AML after MDS/MPN, Overlap/AML with MDS-related changes	59	39	>10	>10	>10
38*	M1	AML without maturation	71	83	0.05	>10	1
42*	M4	AML with NPM1 mutation	57	25	>10	>10	0.76
43*	M4	AML with NPM1 mutation	73	81	>10	>10	0.14
45		AML, NOS	33	74	>10	>10	0.1
47*	M1	AML, NOS	74	71	>10	>10	>10
48	M4	AML with MDS-related changes	58	40	>10	>10	>10
55		secondary AML after MDS	79	25	2.2	9	1.1
56	M4	secondary AML after MDS	75	25	0.05	4	2.5
59*	M1	AML with recurrent genetic modifications	76	71	>10	>10	0.21
60	M5	AML, NOS	22	95	0.07	>10	0.007
**Cell lines**							
MOLM-13	M5a				0.083	>10	0.05
ML-2	M4				0.11	6.8	0.31
HL-60	M2				3	>10	1.98
KG-1	−				4	>10	0.49
MV4-11	M5				0.013	3.3	0.02
KASUMI-1	M2				0.77	1.8	0.15
NB4	M3				3	>10	0.06
PLB985	M2				3	>10	0.22
THP-1	M5				3	>10	0.1
MONO-MAC-6	M5				4	5.6	0.072
OCI-AML3	M4				>10	>10	1.5

EC_50_ values to BH3-mimetics were calculated from combined Annexin-V/FITC and anti-CD45/APC staining and flow cytometry (for primary cells) and CellTiter-Glo viability assay (for cell lines) by non-linear regression analysis using GraphPad prism. For primary AML samples, data were derived from either freshly isolated AML blasts or from previously frozen samples (*). *FAB* French-American-British classification system, *NOS* Not Otherwise Specified, *MDS* Myelodysplastic syndrome, *ND* Not determined

Due to the function of BCL-2 proteins in normal hematopoietic cells, BH3-mimetics may exert substantial toxicities. To assess the effect of BH3-mimetics on normal CD34+ progenitor cells the effect of ABT-199, A1331852 and S63845 on apoptosis and colony formation of purified CD34+ cells was investigated. While both ABT-199 and A1331852 induced apoptosis at submicromolar concentrations in normal CD34+ cells, the MCL-1 inhibitor S63845 only induced apoptosis at high concentrations (Fig. S2a). Also, analysis of colony-forming units showed that colony formation was most affected by A1331852, whereas S63845 and ABT-199 did not result in reduced colony formation (Fig. S2b, c). Taken together, these data indicate a favorable toxicity profile of S63845 as compared to ABT-199 or A1331852 on normal CD34+ progenitor cells.

To better understand the molecular mechanisms underlying the response to BH3-mimetics, we next turned to a panel of AML cell lines (Fig. 1b and Table 1). Four out of eleven cell lines responded to ABT-199 with an EC_50_ of < 3 μM (MV4-11, MOLM-13, ML2, and KASUMI-1) and only one out of 11 cell lines responded to the BCL-x_L_ inhibitor (KASUMI-1), whereas all eleven cell lines responded to the MCL-1 inhibitor S63845. Notably, five cell lines displayed a very high sensitivity to S63845 with an EC_50_ of < 150 nM (MV4-11, MOLM-13, NB4, MONO-MAC-6, and KASUMI-1), and overall S63845 induced significantly more cell death than ABT-199 also in this panel of cell lines (Fig. S1). Induction of apoptosis by these selective BH3-mimetics was confirmed in selected sensitive cell lines as assessed by externalization of phosphatidylserine (PS), staining with Annexin-V/FITC and flow cytometry (Fig. S3). These data indicate that amongst the antiapoptotic BCL-2 proteins, MCL-1 may represent the most prevalent therapeutic target in AML, followed by BCL-2. Interestingly, some cell lines were sensitive to more than one BH3-mimetic (e.g. MOLM-13 and MV4-11 displayed sensitivity to ABT-199 and S63845), indicating that the different antiapoptotic BCL-2 proteins may be functionally redundant. Of note, all cell lines in our panel that responded to ABT-199 reacted to S63845 as well. To investigate whether S63845 and ABT-199 may act together to induce cell death we combined these BH3-mimetics and observed synergistic cell death in MOLM-13 and MONO-MAC-6 cells (Fig. S4).

### Response to BH3-mimetics is independent of BCL-2 protein expression

**Fig. 2:**
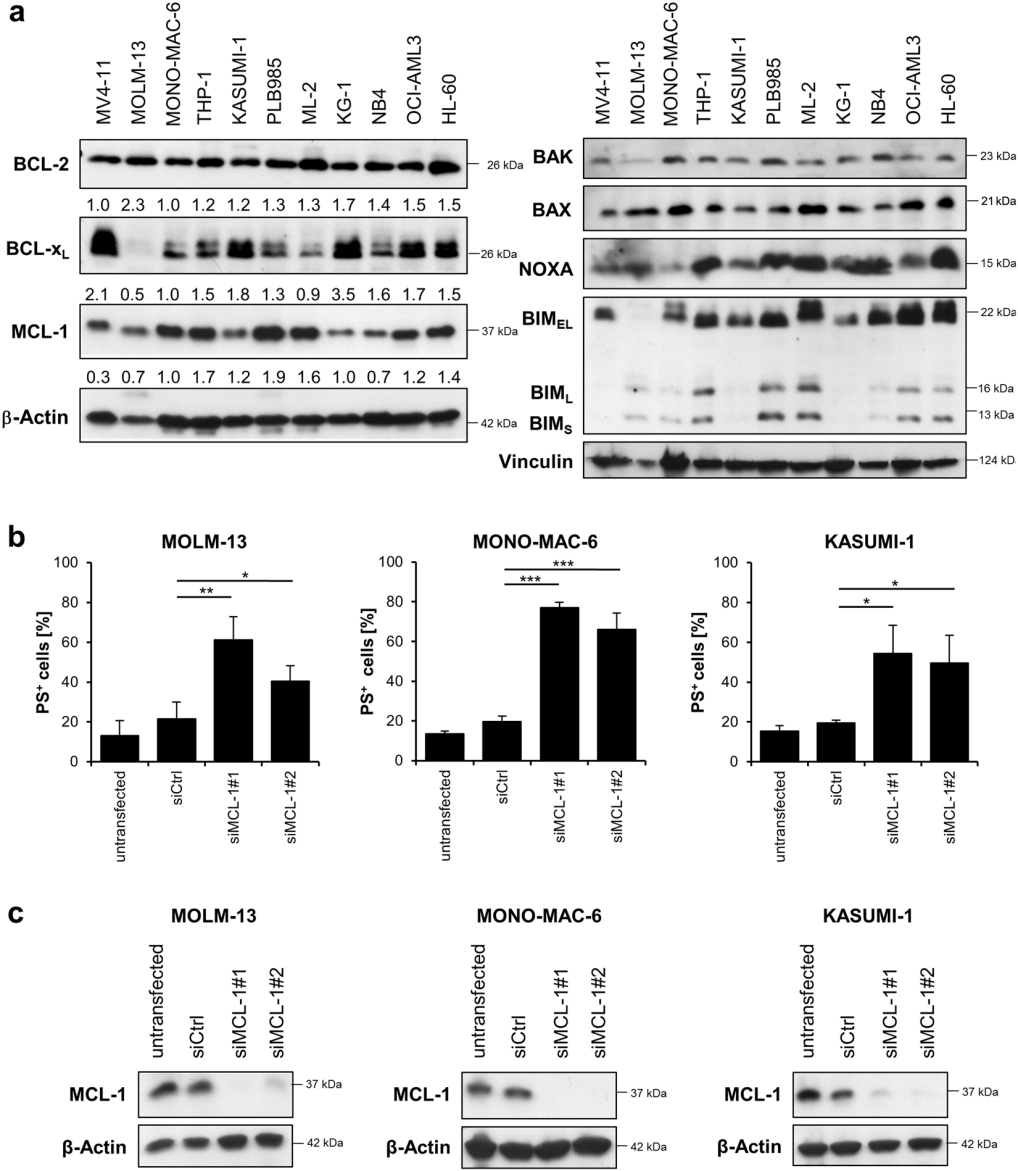
Sensitivity towards BH3-mimetics does not correlate with BCL-2 protein expression levels. **a** Expression levels of the main antiapoptotic (left) or proapoptotic (right) BCL-2 proteins in AML cell lines were analyzed by Western blotting. β-Actin and Vinculin served as loading control. Numbers below the Western blots indicate quantified expression levels of three independent experiments relative to loading control. **b**, **c** Selected AML cell lines were transiently transfected with siRNA against MCL-1 or non-targeting control siRNA (siCtrl). **b** Cell death was determined by externalization of phosphatidylserine (PS) and flow cytometry at 24 h after transfection in MOLM-13, MONO-MAC-6 or KASUMI-1 cells. Data are mean and standard deviation (SD) (*n* = 3). *P < 0.05; **P < 0.01; ***P < 0.001. **c** MCL-1 expression was assessed by Western blotting with β-Actin serving as loading control.

The response to BH3-mimetics may be associated with expression levels of BCL-2 proteins. Western blotting revealed that the main antiapoptotic BCL-2 proteins BCL-2, BCL-x_L_ and MCL-1 were expressed in all AML cell lines studied, with BCL-2 being expressed at comparable levels in all cell lines (Fig. 2a). Linear regression analysis confirmed that expression of BCL-2 did not correlate with susceptibility to ABT-199 (Fig. S5), as also observed in previous studies^9,23^. Expression levels of BCL-x_L_ and MCL-1 varied between the cell lines, the expression of BCL-x_L_ being highest in MV4-11 cells and lowest in MOLM-13 cells. The cell line that was most sensitive to A1331852, KASUMI-1, expressed moderate levels of BCL-x_L_, demonstrating that the response to A1331852 did not correlate with expression levels of its target BCL-x_L_. Expression of MCL-1 or BCL-x_L_ also did not associate with susceptibility to S63845. A previous study has suggested an inverse correlation between susceptibility to S63845 and expression of BCL-x_L_^19^, but the most sensitive cell lines MOLM-13, MV4-11, NB4, MONO-MAC-6, and KASUMI-1 did not express less BCL-x_L_ than the less sensitive cell lines in our panel. The susceptibility to BH3-mimetics may depend on the presence of proapoptotic BCL-2 proteins such as BAK and BAX. Both BAK and BAX were expressed in all of the tested cell lines, indicating that lower susceptibility to BH3-mimetics is not mediated by the absence of BAX or BAK (Fig. 2a). BH3-only proteins like NOXA and BIM were also expressed in AML cells, although the expression levels of BIM varied between the cell lines studied.

To confirm that the MCL-1 inhibitor S63845 displays on-target toxicity and that AML cells indeed depend on MCL-1 for cellular survival we performed genetic silencing of MCL-1 followed by analysis of cell death. Importantly, transient knockdown of MCL-1 using two distinct siRNA sequences was sufficient to induce cell death in MOLM-13, MONO-MAC-6 and KASUMI-1 cells (Fig. 2b). Knockdown efficiency was confirmed by Western blotting (Fig. 2c).

### BH3-mimetics induce rapid caspase cleavage and caspase-dependent apoptosis

**Fig. 3:**
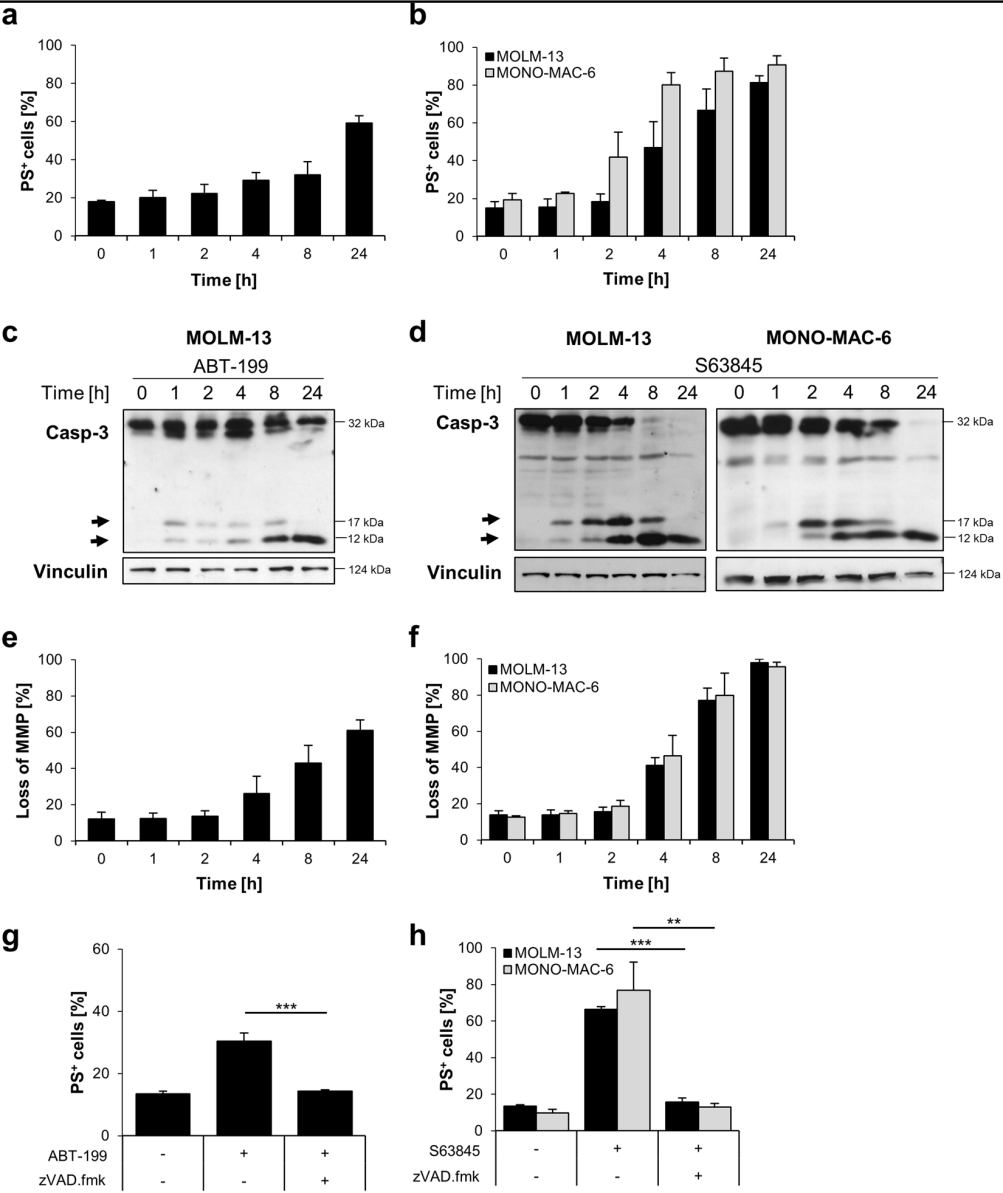
Primary route of cell death induced by BH3-mimetics is via caspase-dependent intrinsic apoptosis. **a** MOLM-13 cells were treated with ABT-199 (1 μM) for indicated time prior to analysis of cell death by Annexin-V/FITC staining and flow cytometry. **b** MOLM-13 or MONO-MAC-6 cells were treated with S63845 (1 μM) for indicated time points prior to the analysis of apoptosis by Annexin-V/FITC staining and flow cytometry. **c**, **d** Caspase-3 cleavage was analyzed by Western blotting. Vinculin served as loading control. Arrowheads indicate cleavage fragments of caspase-3. **e**, **f** Loss of MMP was measured using TMRM staining and flow cytometry upon treatment with 1 μM of ABT-199 e or 1 μM of S63845 **f**–**h**. MOLM-13 or MONO-MAC-6 cells were treated with ABT-199 **g** or S63845 **h** for 4 h in the presence or absence of 50 μM of the pan-caspase inhibitor zVAD.fmk. Cell death was determined by Annexin-V/FITC staining and flow cytometry. Mean and SD of three independent experiments performed in triplicate are shown. **P < 0.01; ***P < 0.001.

To further investigate the mechanism of cell death, representative cell lines were selected that displayed sensitivity to the respective BH3-mimetics. As only the KASUMI-1 cells displayed sensitivity to the BCL-x_L_ inhibitor A1331852, we concentrated the following investigations on a comparison between ABT-199 and S63845. MOLM-13 cells displayed sensitivity to ABT-199 and S63845, whereas MONO-MAC-6 cells only displayed sensitivity to S63845. Cell death induced by ABT-199 was relatively slow in MOLM-13 cells, with PS-exposure only detectable after 8 to 24 h of treatment (Fig. 3a). Interestingly, cell death induced by S63845 was more rapid, with PS-exposure detected after only 4 h of treatment in both MOLM-13 and MONO-MAC-6 cells (Fig. 3b). To investigate whether caspases are cleaved upon treatment with BH3-mimetics, Western blotting was performed. Cleaved caspase-3 was detected already after one hour of treatment. In line with the more rapid cell death induction by S63845 as compared to ABT-199, caspase-3 cleavage was more pronounced after treatment with S63845 and the pro-form of caspase-3 was processed more efficiently into cleaved fragments (Fig. 3c, d). To investigate whether mitochondria were involved in apoptosis induction, the mitochondrial membrane potential (MMP) was analyzed by staining with TMRM (Fig. 3e, f). Cell death was associated with loss of MMP in similar kinetics as PS-exposure. To investigate whether cell death induced by BH3-mimetics resulted in caspase-dependent cell death, we added the caspase inhibitor zVAD.fmk (Fig. 3g, h). Inhibition of caspases resulted in a significant decrease in cell death at 8 h, indicating that the primary route of cell death is via caspase-dependent apoptosis. However, when cell death was measured at 24 h, the caspase inhibitor zVAD.fmk was no longer able to prevent cell death, indicating that if caspases are blocked, cell death can occur via a caspase-independent form of cell death upon prolonged treatment, as we have previously shown for other stimuli^24,25^ (Fig. S6). Taken together, these data indicate that cell death induced by BH3-mimetics was executed primarily via the intrinsic apoptotic pathway, as anticipated by on-target inhibition of BCL-2 proteins.

### ABT-199 induces BAX-dependent apoptosis, while S63845 induces mainly BAK-dependent apoptosis

**Fig. 4:**
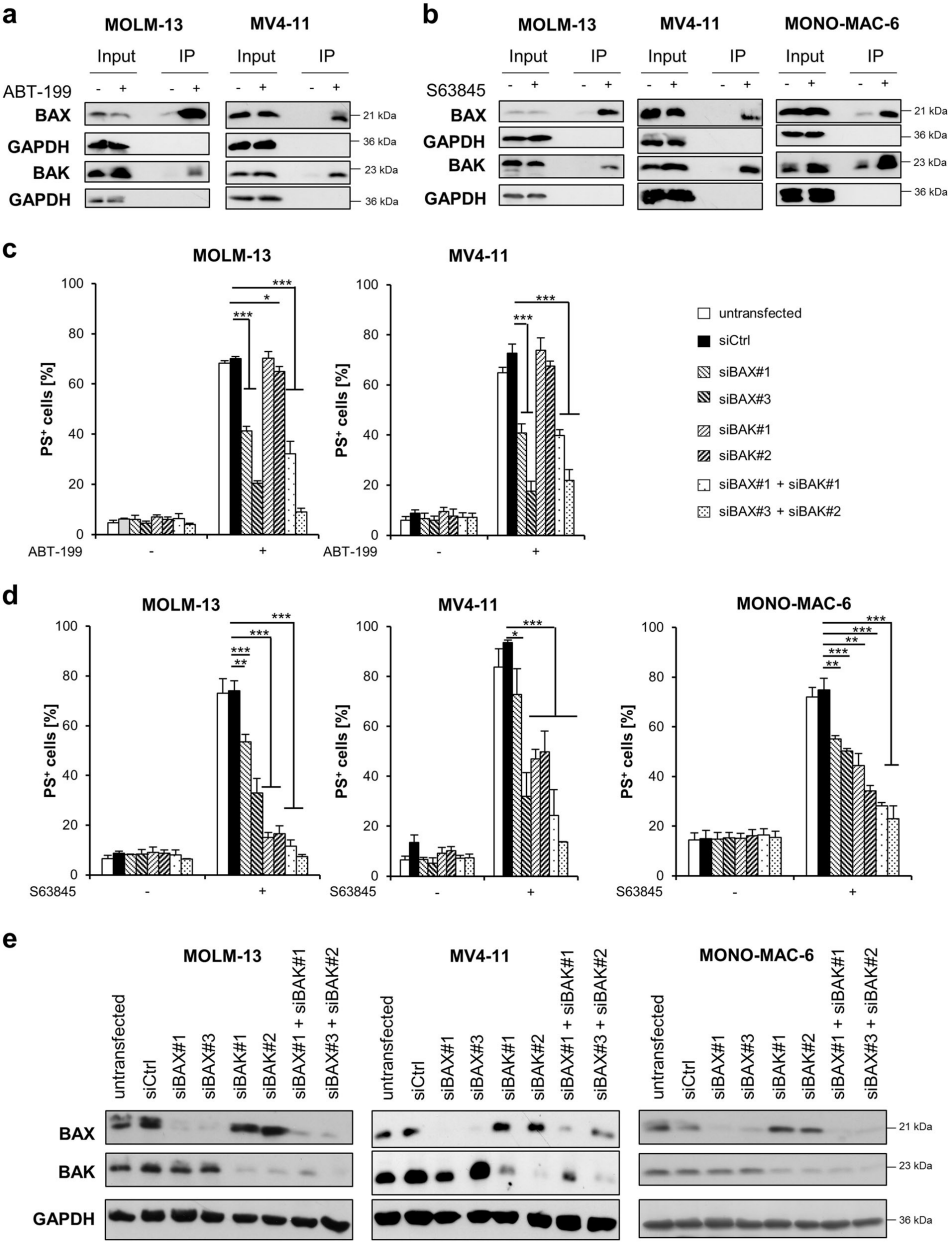
BH3-mimetics induced BAX- or BAK-dependent apoptosis. **a**, **b** MOLM-13 or MV4–11 cells were treated for 8 h with ABT-199 (1 μM) or for 4 h with S63845 (1 μM). MONO-MAC-6 cells were treated with S63845 (1 μM) for 2 h prior to analysis of BAX and BAK activation by IP. Input is shown to demonstrate equal protein expression in the lysate. A representative experiment is shown (*n* = 2–3). **c**, **d** MOLM-13, MV4-11 or MONO-MAC-6 cells were transiently transfected with siRNA against BAX or BAK or non-targeting control siRNA (siCtrl), followed by treatment with **c** ABT-199 (24 h) or **d** S63845 (MOLM-13 and MV4-11: 4 h, MONO-MAC-6: 2 h) and analysis of apoptosis by staining with Annexin-V/FITC and flow cytometry. Mean and SD of three independent experiments performed in triplicate are shown. *P < 0.05; **P < 0.01; ***P < 0.001. **e** Expression levels of BAX and BAK were assessed by Western blotting for transfected MOLM-13, MV4-11 and MONO-MAC-6 cells with GAPDH serving as loading control.

Loss of MMP and release of cytochrome *c* are mediated by the proapoptotic BCL-2 proteins BAX and BAK^26^. During their activation BAX and BAK undergo conformational changes including the exposure of new domains in the N-terminal protein^27,28^. To investigate whether BAK and/or BAX are activated upon treatment with BH3-mimetics, we performed immunoprecipitations with specific antibodies against the N-terminal domains exposed during activation of BAX and BAK^29^. Of note, treatment with BH3-mimetics induced activation of both BAX and BAK in MOLM-13, MV4-11 and MONO-MAC-6 cells (Fig. 4a, b), as well as in primary AML cells (Fig. S7). Next, we asked whether BAX and/or BAK were required for apoptosis induced by ABT-199 or S63845. To this end, we performed silencing of BAK or BAX using two distinct siRNA oligonucleotides for either BAX or BAK. Interestingly, knockdown of BAX inhibited ABT-199-induced apoptosis in MOLM-13 and MV4-11 cells, whereas knockdown of BAK had no effect (Fig. 4c). Notably, double knockdown of BAX and BAK did not prevent apoptosis substantially more than single knockdown of BAX. In contrast, in MOLM-13, MV4-11 and MONO-MAC-6 cells, S63845-induced apoptosis was more dependent on BAK than on BAX, and knockdown of BAK was able to almost completely inhibit S63845-induced apoptosis in MOLM-13 cells (Fig. 4d). Knockdown efficiency was confirmed by Western blotting (Fig. 4e). These data indicate that, upon inhibition of MCL-1, apoptosis is induced in a predominately BAK-dependent manner, whereas inhibition of BCL-2 induces activation of BAX-dependent apoptosis.

### BH3-mimetics displace proapoptotic BH3-containing proteins from their antiapoptotic target

**Fig. 5:**
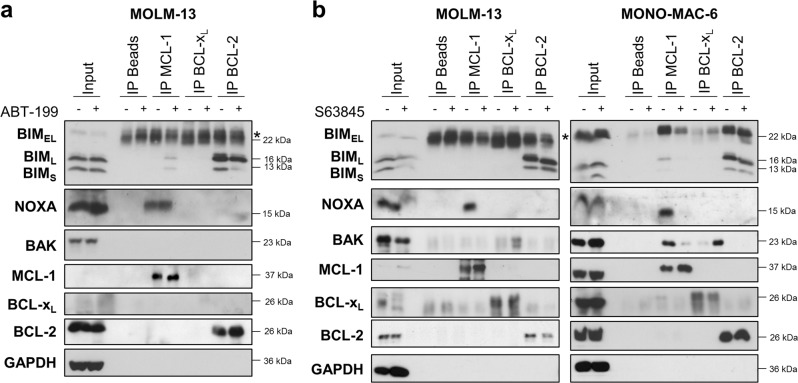
BH3-mimetics displace proapoptotic BCL-2 proteins from antiapoptotic targets. **a** MOLM-13 cells were treated with 1 μM ABT-199 for 8 h. **b** MOLM-13 or MONO-MAC-6 cells were treated with 1 µM S63845 for 4 or 2 h, respectively. **a**, **b** MCL-1, BCL-x_L_ and BCL-2 were immunoprecipitated followed by analysis of proapoptotic binding partners by Western blotting with GAPDH serving as loading control. The input is shown for basal protein expression levels. * indicates IgG band.

To further investigate how BAX and BAK might be activated upon treatment with BH3-mimetics, we investigated the interactions between pro- and antiapoptotic BCL-2 proteins. Using immunoprecipitation of MCL-1, BCL-x_L_, and BCL-2, we found that the proapoptotic BH3-only protein BIM was primarily bound to BCL-2 in MOLM-13 cells and to BCL-2 and MCL-1 in MONO-MAC-6 cells (Fig. 5). Binding of BIM to BCL-x_L_ was hardly detectable in both cell lines. NOXA was exclusively bound to MCL-1. Thus, these cells constitutively display a high level of priming and MCL-1, as well as BCL-2 function to sequester BH3-only proteins. Treatment with ABT-199 did not influence the binding of NOXA to MCL-1 but displaced some sequestered BIM_L_ and BIM_S_ from BCL-2, which then appeared to be bound by MCL-1 (Fig. 5a, Fig. S8a). The MCL-1 inhibitor S63845 displaced BIM from MCL-1 in MONO-MAC-6 cells, while it did not affect BIM binding to BCL-2 in MOLM-13 or MONO-MAC-6 cells (Fig. 5b, Fig. S8b). In both cell lines, treatment with S63845 displaced NOXA from MCL-1, demonstrating on-target activity of S63845 (Fig. 5b). Notably, in MONO-MAC-6 cells we also observed a direct sequestration of BAK by MCL-1. Intriguingly, treatment with S63845 disrupted the binding of BAK to MCL-1, and the released BAK was then bound by BCL-x_L_ (Fig. 5b).

**Fig. 6:**
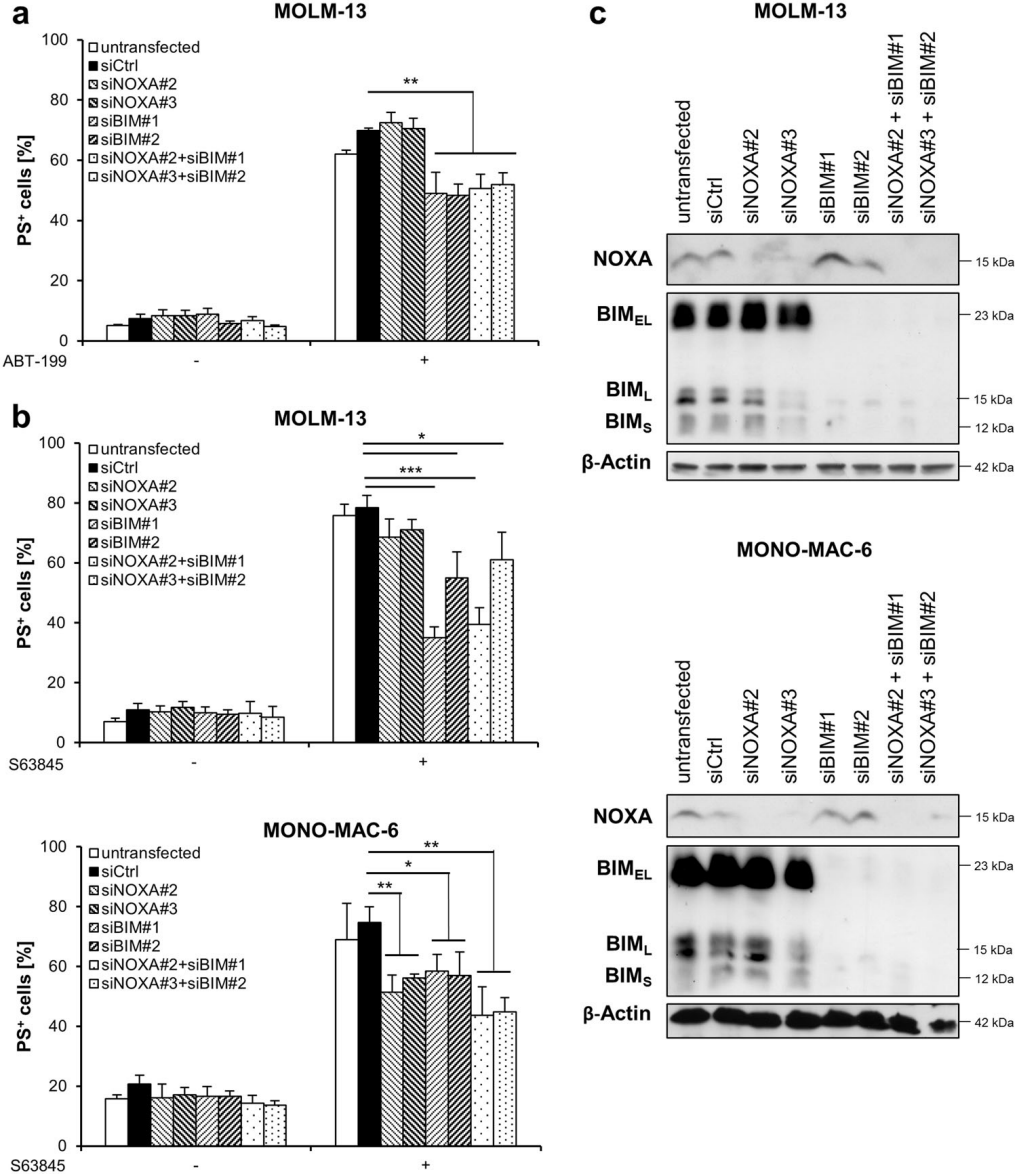
BH3-mimetic-induced apoptosis is partially BIM-dependent. **a** MOLM-13 cells were transiently transfected with siRNA against NOXA, BIM or non-targeting control siRNA (siCtrl), followed by treatment with ABT-199 for 24 h and analysis of apoptosis by staining with Annexin-V/FITC and flow cytometry. **b** MOLM-13 or MONO-MAC-6 cells were transiently transfected with siRNA against NOXA, BIM or non-targeting control siRNA (siCtrl), followed by treatment with S63845 for 4 h (MOLM-13) or 2 h (MONO-MAC-6) and analysis of apoptosis by staining with Annexin-V/FITC and flow cytometry. Mean and SD of at least three independent experiments performed in triplicate are shown. *P < 0.05; **P < 0.01; ***P < 0.001. **c** Expression levels of NOXA and BIM were assessed by Western blotting for transfected MOLM-13 and MONO-MAC-6 cells with β-Actin serving as loading control.

To investigate whether the displacement of BH3-only proteins was important for BH3-mimetic-induced apoptosis we performed siRNA-mediated knockdown of BIM and/or NOXA using two distinct siRNA oligonucleotides. Loss of BIM had a minor but significant effect on ABT-199-induced apoptosis in MOLM-13 cells, whereas NOXA was not involved in ABT-199-induced apoptosis (Fig. 6a). By comparison, loss of BIM more substantially inhibited S63845-induced apoptosis in MOLM-13 cells and to a lesser extent in MONO-MAC-6 cells (Fig. 6b). In the MONO-MAC-6 cells, NOXA also contributed to S63845-induced apoptosis. Double knockdown of BIM and NOXA did not significantly alter apoptosis as compared to single knockdown, indicating that these two BH3-only proteins do not functionally compensate the loss of either protein. Knockdown efficiency was controlled by Western blotting (Fig. 6c). In conclusion, these data indicate that BH3-mimetic-induced apoptosis was partially dependent on the BH3-only protein BIM and in the case of S63845 also on NOXA, but that loss of BH3-only proteins is not sufficient to completely prevent apoptosis.

## Discussion

This study provides the first side-by-side comparison of selective and potent BH3-mimetics targeting the main antiapoptotic proteins MCL-1, BCL-2 or BCL-x_L_ in a larger panel of AML primary samples and AML cell lines. Previous studies have focused on either BCL-2^11^ or MCL-1^18,19^ as therapeutic targets in AML. However, with the arrival of these compounds in clinical applications, it is essential to directly compare the potential of targeting these antiapoptotic BCL-2 proteins.

Our study revealed that the MCL-1 inhibitor S63845 was more potent than the BCL-2 inhibitor ABT-199 both in primary cells and derived cell lines. This difference cannot be easily explained by different binding potencies of these compounds to their respective target, as all three compounds tested in this study have subnanomolar binding affinities and display low nanomolar cytotoxicity in other cellular systems^14,19^. A more prominent role of MCL-1 as compared to BCL-2 in AML is supported by genetic studies in which the deletion of MCL-1 but not BCL-2 or BCL-x_L_ was shown to cure AML in animal models^18^. In contrast to MCL-1 and BCL-2, we found that although BCL-x_L_ was expressed at high levels, it did not represent a prevalent therapeutic target in AML, with only one sensitive cell line (KASUMI-1) identified here. However, this neither rules out the possibility that BCL-x_L_ may be involved in acquired resistance to BCL-2 or MCL-1 inhibitors, as shown previously for ABT-199^10^, nor excludes an important role of BCL-x_L_ under certain cellular conditions, as suggested for polyploidy resistance^30^.

By demonstrating a heterogeneous response to BH3-mimetics in AML, our study highlights the need to better understand the molecular mechanisms conferring sensitivity in order to identify predictive biomarkers. In contrast to a recent study^31^, we did not observe a relationship between *NPM1* mutations and sensitivity to ABT-199, as the three patients’ samples with *NPM1* mutation were all resistant to ABT-199. Similarly we did not observe an inverse correlation of S63845 sensitivity and the expression levels of BCL-x_L_, indicating that other factors may play a role in conferring resistance to S63845 in AML^19^. Notably, while the previous study had assessed BCL-x_L_ mRNA expression^19^, our analysis was based on protein expression.

S63845-induced cell death was not only more commonly observed, but also occurred faster than ABT-199-induced cell death, pointing to a molecular difference in the signaling events induced by inhibition of MCL-1 and BCL-2, respectively. This hypothesis is supported by our findings that ABT-199-induced apoptosis was mediated by BAX, whereas S63845-induced apoptosis was mainly BAK-dependent. Further evidence showing that ABT-199-induced cell death is mediated by BAX was provided by a study that described BAX-dependent cell death in MOLM-13 and MV4-11 cells following combined treatment with ABT-199 and PI3K inhibitors^32^. S63845-induced apoptosis has previously been shown to be BAK- but not BAX-dependent in breast cancer cells^33^. Here, we find that in the MCL-1-dependent MONO-MAC-6 cells, BAK was constitutively sequestered by MCL-1, while we did not detect any direct sequestration of BAX. This confirms the observation that, particularly in hematopoietic cells, BAK but not BAX is endogenously oligomerized and sequestered by antiapoptotic BCL-2 proteins^34^. Of note, we found BAK sequestered by MCL-1 more than by BCL-x_L_, which may explain the importance of MCL-1 in maintaining survival and indicate a potential biomarker for S63845 sensitivity that is independent of BCL-x_L_ expression levels. Upon treatment with S63845, BAK was displaced from MCL-1 and now bound more strongly to BCL-x_L_, but not to BCL-2, supporting a potential role of BCL-x_L_ in compensating for MCL-1 inhibition upon acquired resistance.

The BH3-only protein BIM was bound to BCL-2 in MOLM-13 and to BCL-2 and MCL-1 in MONO-MAC-6 cells, whereas NOXA was bound to MCL-1 in both cell lines. Treatment with S63845 displaced BIM and NOXA from MCL-1, while treatment with ABT-199 displaced BIM from BCL-2, confirming on-target activity of these BH3-mimetics. Notably, our data also indicate that the displaced BIM is now bound by other antiapoptotic BCL-2 proteins, providing a mechanistic explanation of the synergy observed between ABT-199 and S63845. Displacement of BIM from BCL-2 by ABT-199 and subsequent binding by MCL-1 were previously suggested as a mechanism of resistance to ABT-199^35^. However, several reports have shown that BIM is not required for BH3-mimetic-induced apoptosis^32,36^. In line with this model, we detected only a moderate reduction of BH3-mimetic-induced apoptosis upon BIM knockdown. Similarly, knockdown of NOXA resulted in a reduction of S63845-induced cell death particularly in the MONO-MAC-6 cells. These data indicate that BIM and NOXA may partially contribute to BH3-mimetic-induced cell death in AML cells. BIM has frequently been described as direct activator of BAX and BAK, and similarly, NOXA may under certain conditions act as a weak direct activator^37^, thus providing a mechanistic explanation as to how BH3-only proteins could contribute to BH3-mimetic-induced apoptosis. In addition, BH3-mimetics may induce activation of BAX/BAK independently of any BH3-only proteins by binding to and occupying antiapoptotic BCL-2 proteins on the OMM, which may directly increase BAX/BAK localization to the OMM and their subsequent activation^38^. In this model, unbound antiapoptotic BCL-2 proteins are essential in preventing BAX/BAK insertion into the OMM, and binding of BH3-mimetics and/or BH3-only proteins to the antiapoptotic BCL-2 proteins may neutralize their ability to prevent BAX/BAK insertion.

Taken together, the direct comparison of selective BH3-mimetics in AML cells reveals S63845 to have higher potency against primary AML cells and derived cell lines than ABT-199 or A1331852. In addition, we find S63845 to be less toxic against healthy CD34+ progenitor cells, indicating that S63845 may have a favorable therapeutic window in comparison with ABT-199. On a molecular level, MCL-1 functions by sequestering BIM, NOXA and BAK. Treatment with S63845 releases or displaces these proapoptotic binding partners from MCL-1, leading to mainly BAK-dependent apoptosis.

## Materials and methods

### Cell culture

ML-2, THP-1 and PLB985 cells were kindly provided by T. Oellerich, Hematology/Oncology, Goethe University, Frankfurt, Germany. All other cell lines were obtained from German Collection of Microorganisms and Cell cultures (DSMZ, Braunschweig, Germany). All cell lines were authenticated by STR-profiling at the DSMZ and routinely checked for mycoplasma contamination. All cell lines except KASUMI-1, MONO-MAC-6 and OCI-AML3 were cultured in RPMI 1640 medium (Life Technologies, Inc., Darmstadt, Germany) supplemented with 10% fetal calf serum (FCS), 1% penicillin/streptomycin and 1% sodium pyruvate (Invitrogen, Karlsruhe, Germany). KASUMI-1 cells were cultured in RPMI 1640 supplemented with 20% FCS, 1% penicillin/streptomycin and 1% sodium pyruvate, OCI-AML3 cells were cultured in alpha-MEM medium supplemented with 20% FCS, 1% penicillin/streptomycin and 1% sodium pyruvate and MONO-MAC-6 cells were cultured in RPMI 1640 supplemented with 10% FCS, 1% non-essential amino acids, 1% penicillin/streptomycin, 1% sodium pyruvate and 10 µg/ml insulin. All cell lines were incubated at 37 °C with 5% CO_2_.

### Primary AML samples

Peripheral blood and bone marrow specimens from AML patients were obtained from the UCT Biobank (University Hospital Frankfurt) with written informed consent and the approval of the local ethics committee (SH-05-2014). Mononuclear cell (MNC) fractions were obtained by density gradient centrifugation using Ficoll Isopaque (Amersham Bioscience, Freiburg, Germany) and maintained as previously described^39^. Primary samples with spontaneous apoptosis ≥ 40% at the time point of measurement were excluded from the analysis. MNCs and clinical data were provided by UCT Biobank. Primary AML patient-derived cells were seeded in a density of 1 × 10^6^ cells/ml and treated with BH3-mimetics. After 20 h cells were stained with Annexin-V/FITC and 0.5 μl anti-CD45/APC antibody (eBioscience, 17-0459-42). Cell death was determined by flow cytometry (FACSCanto II, BD Biosciences, Heidelberg, Germany) with a gate on the AML blast population identified by CD45/SSC analysis as described previously^40^.

### CellTiter-Glo viability assay

The sensitivity of AML cell lines towards ABT-199 (Selleck), A1331852 (Selleck) and S63845 (Appexbio) was assessed with CellTiter-Glo viability assay (Promega). Cells were seeded in a density of 1 × 10^5^ cells/ml in a white 96-well plate. After 72 h of incubation 5 μl/well of CellTiter-Glo reagent was added and luminescence was measured with a Tecan Infinite M200 plate reader. Values were normalized to the untreated control sample.

### Cell death analysis

Cells were seeded in a density of 2 × 10^5^ cells/ml in a 96-well plate and treated with BH3-mimetics for indicated time points. Afterwards, cells were incubated with Annexin-V/FITC prior to analysis of cell death by flow cytometry. To investigate the role of caspases, zVAD.fmk (50 μM) (Bachem) was added to the cells. To measure changes in MMP upon treatment with BH3-mimetics, tetramethylrhodamine methyl ester (50 nM) (TMRM Reagent; Thermo Fisher) was added for 10 min at 37 °C followed by flow cytometry.

### Western blot analysis

Protein extraction was done with TritonX-containing lysis buffer and protein content was determined using BCA assay (Pierce^TM^ BCA protein assay, Thermo Fisher Scientific). SDS–PAGE was carried out followed by semidry blotting. After blocking the membrane for 1 h with 5% milk, proteins were detected using the following antibodies: rabbit anti-MCL-1 (Enzo, ADI-AAP-240F), mouse anti-BCL-2 (Dako (Agilent), M088701-2), rabbit anti-BCL-x_L_ (CellSignaling, 2762 S), mouse anti-NOXA (Enzo, ALX-804-408), mouse anti-BAX (BD Bioscience, 610983), rabbit anti-BAK (Upstate/ Merck, 06–536), rabbit anti-BIM (CellSignaling, 3183 S) and rabbit anti-caspase-3 (CellSignaling, 9662 S), mouse anti-β-Actin (Sigma, A5441), mouse anti-GAPDH (BioTrend (Hy Test Ltd), 5G4-6C5) or mouse anti-Vinculin (Sigma/Merck, V9131-100UL). Quantification of protein expression was performed using ImageJ 3.1 software.

### Immunoprecipitation (IP)

Cells were lysed with CHAPS lysis buffer (containing 10 mM HEPES, 150 mM NaCl, 1% CHAPS (Sigma), pH 7.4) supplemented with protease inhibitor cocktail (Roche, 1169749800). To measure conformational changes associated with the activation of BAX and BAK mouse anti-BAX clone 6A7 (Sigma) or mouse anti-BAK AB-1 (Calbiochem) were used as described previously^29^. For analysis of interactions within the BCL-2 family, mouse anti-MCL-1 (BD Bioscience, 559027), hamster anti-BCL-2 (BD Bioscience, 551051) and rabbit anti-BCL-x_L_ (Abcam, ab32370) were used upon crosslinking with Dyna protein G beads (Thermo Fisher, 10004D) using 20 mM dimethyl pimelimidate (Sigma).

### RNA interference

Gene silencing was performed using Neon Transfection System (Invitrogen) following the manufacturer’s instructions. Cells were transfected with 100 nM of targeting Silencer Select siRNA for MCL-1 (#1: s8583, #2: s8585) or non-targeting control siRNA (4390843). For knockdown of BAX, BAK, BIM or NOXA, cells were transfected twice with siRNA targeting BAX (#1: s1888, #3: s1890), BAK (#1: s1880, #2: s1881), NOXA (#2: s10709, #3: s10710) or BIM (#1: s190511, #2: s195012). 48 h after transfection, cells were treated with ABT-199 (24 h) or S63845 (2 or 4 h) before analysis of cell death by staining with Annexin-V/FITC and flow cytometry.

### Statistics

EC_50_ values were calculated with non-linear regression algorithms in GraphPad Prism software. Statistical significance was verified by using *t*-test in Excel (two-samples, two-tailed distribution, unequal variance). The numbers of independent repetitions and replicates for each experiment are indicated in the respective figure legends. Experiments were considered as reliable, if the SD did not exceed 10% within the replicates and repetitions.

## Supplementary information

Supplementary Figure 1

Supplementary Figure 2

Supplementary Figure 3

Supplementary Figure 4

Supplementary Figure 5

Supplementary Figure 6

Supplementary Figure 7

Supplementary Figure 8

Supplementary Figure Legends
