# Supplementary figures and images for: Side-by-side comparison of BH3-mimetics identifies MCL-1 as a key therapeutic target in AML
Source: Cell Death Dis. 2019 Dec 4;10(12):917. doi: 10.1038/s41419-019-2156-2 (PMC6892884; doi:10.1038/s41419-019-2156-2)

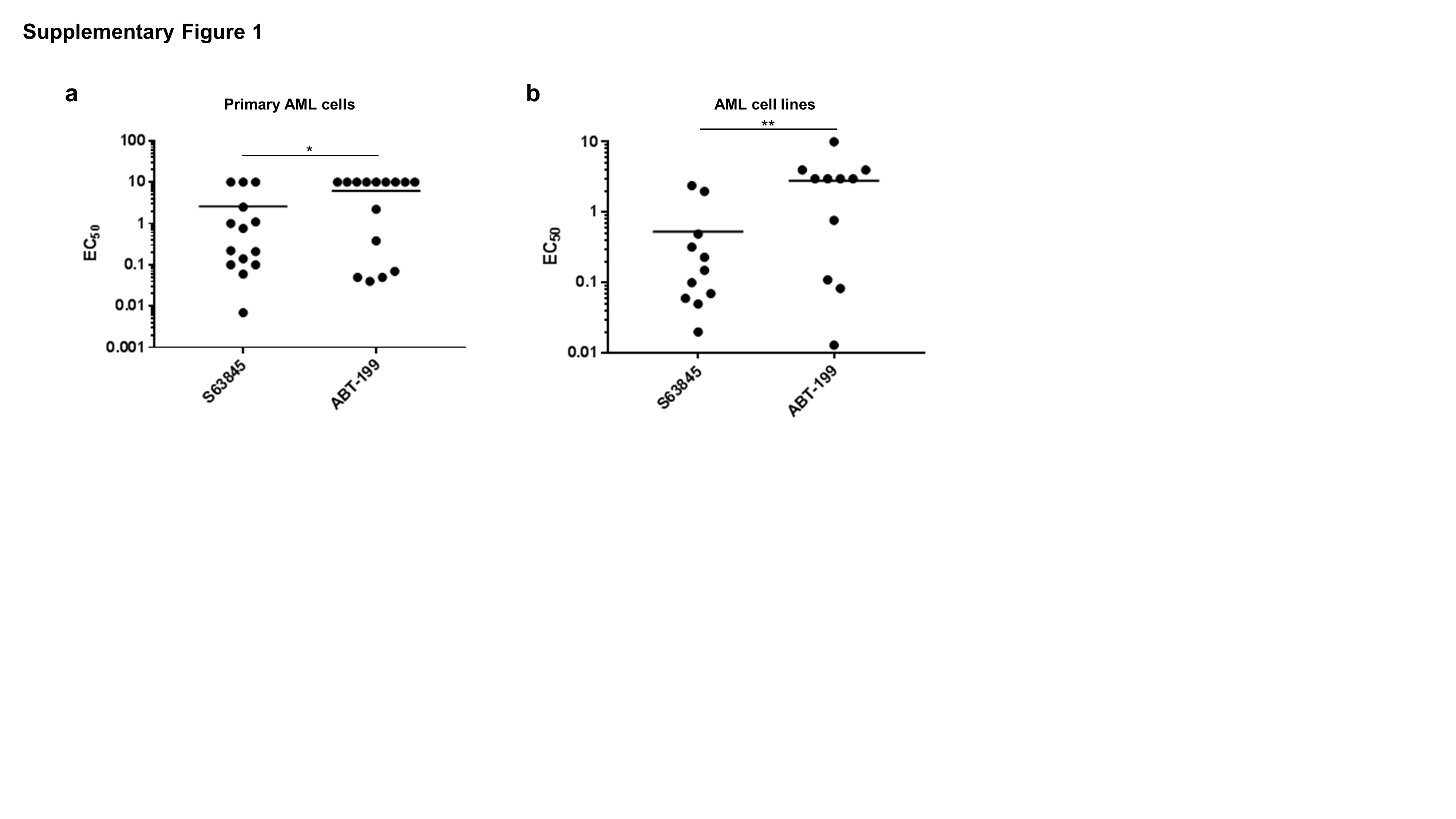

Supplement: Supplementary file 1 — Supplementary Figure 1 [file 41419_2019_2156_MOESM1_ESM.tif]

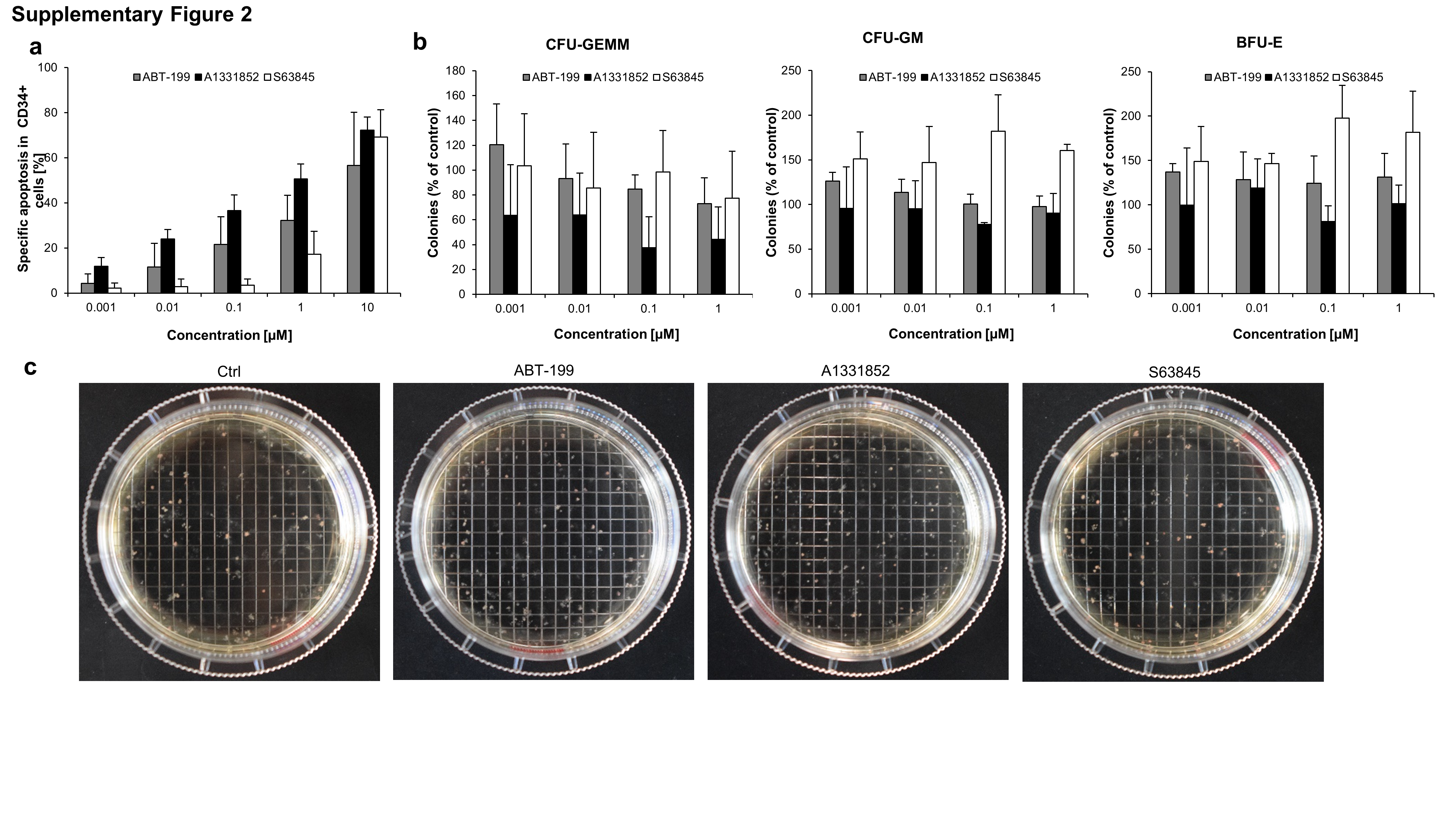

Supplement: Supplementary file 2 — Supplementary Figure 2 [file 41419_2019_2156_MOESM2_ESM.tif]

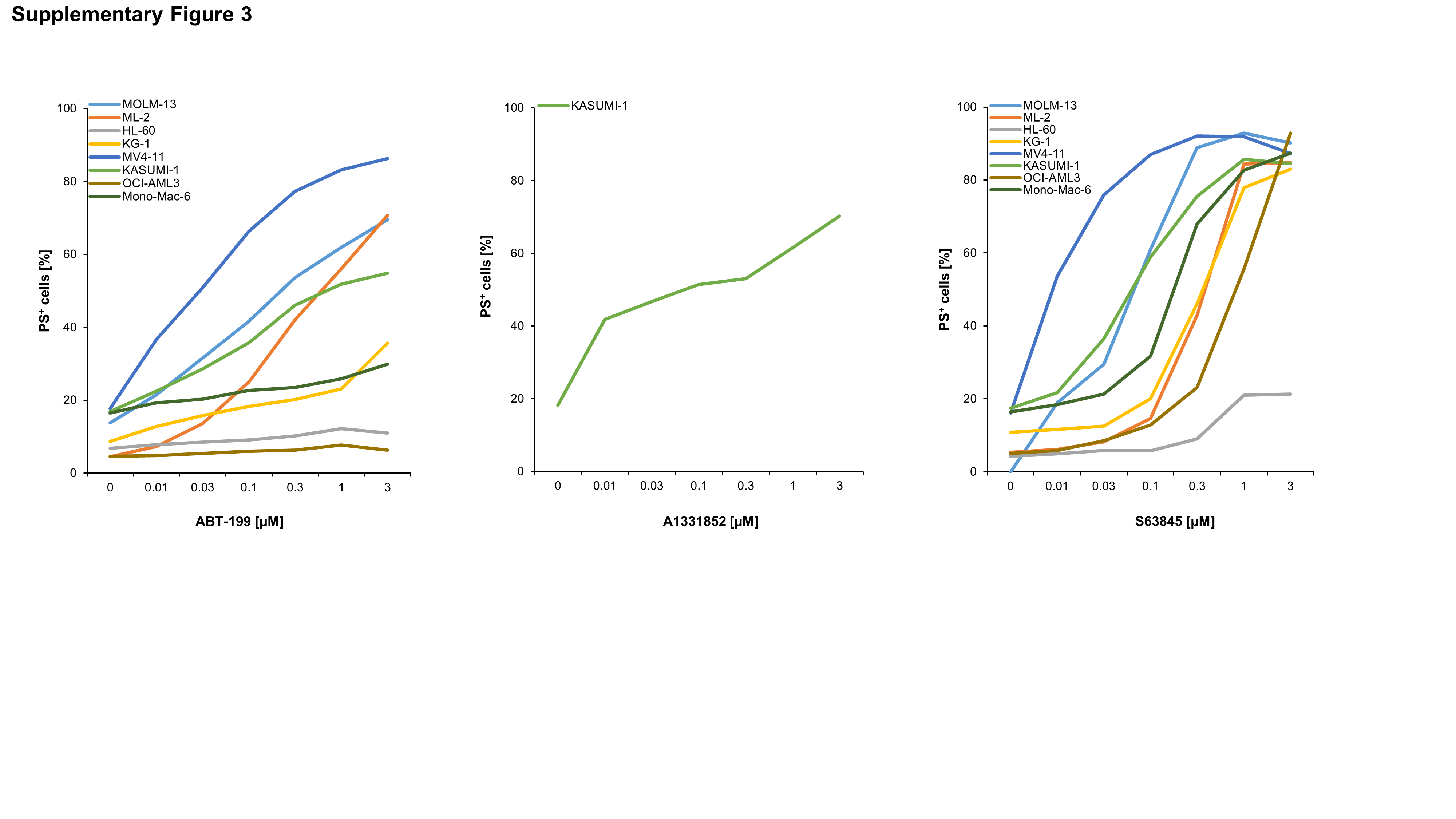

Supplement: Supplementary file 3 — Supplementary Figure 3 [file 41419_2019_2156_MOESM3_ESM.tif]

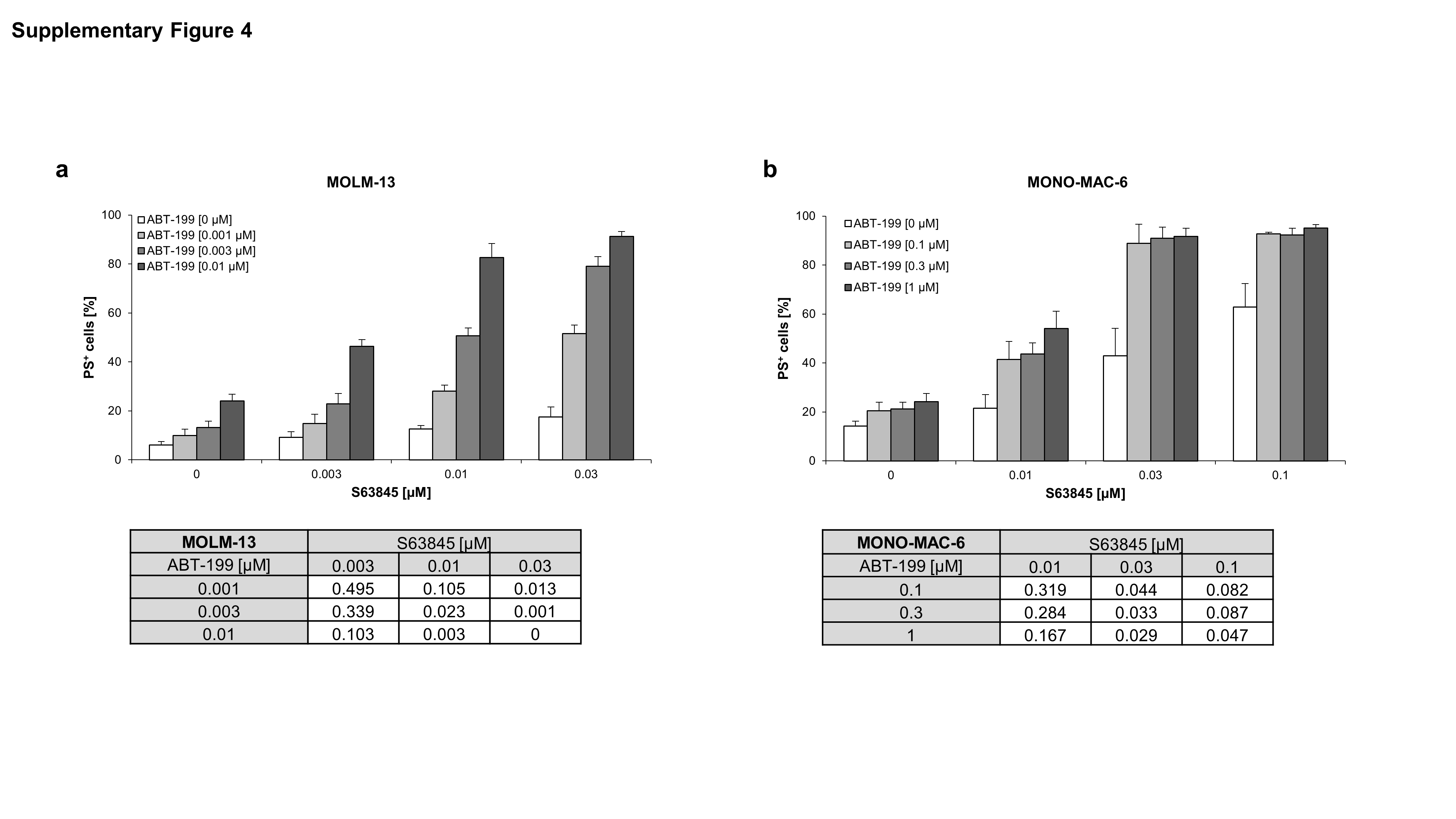

Supplement: Supplementary file 4 — Supplementary Figure 4 [file 41419_2019_2156_MOESM4_ESM.tif]

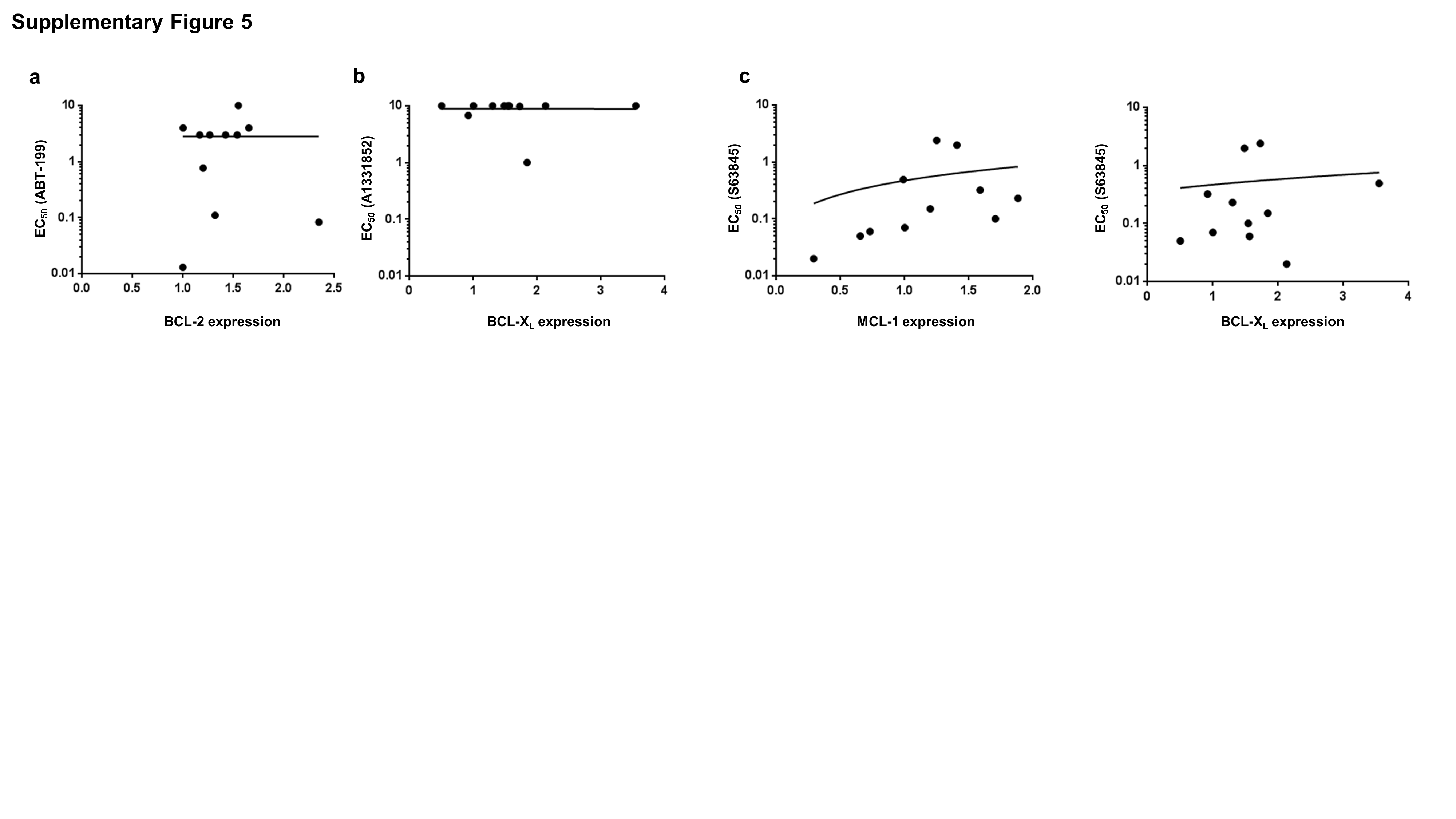

Supplement: Supplementary file 5 — Supplementary Figure 5 [file 41419_2019_2156_MOESM5_ESM.tif]

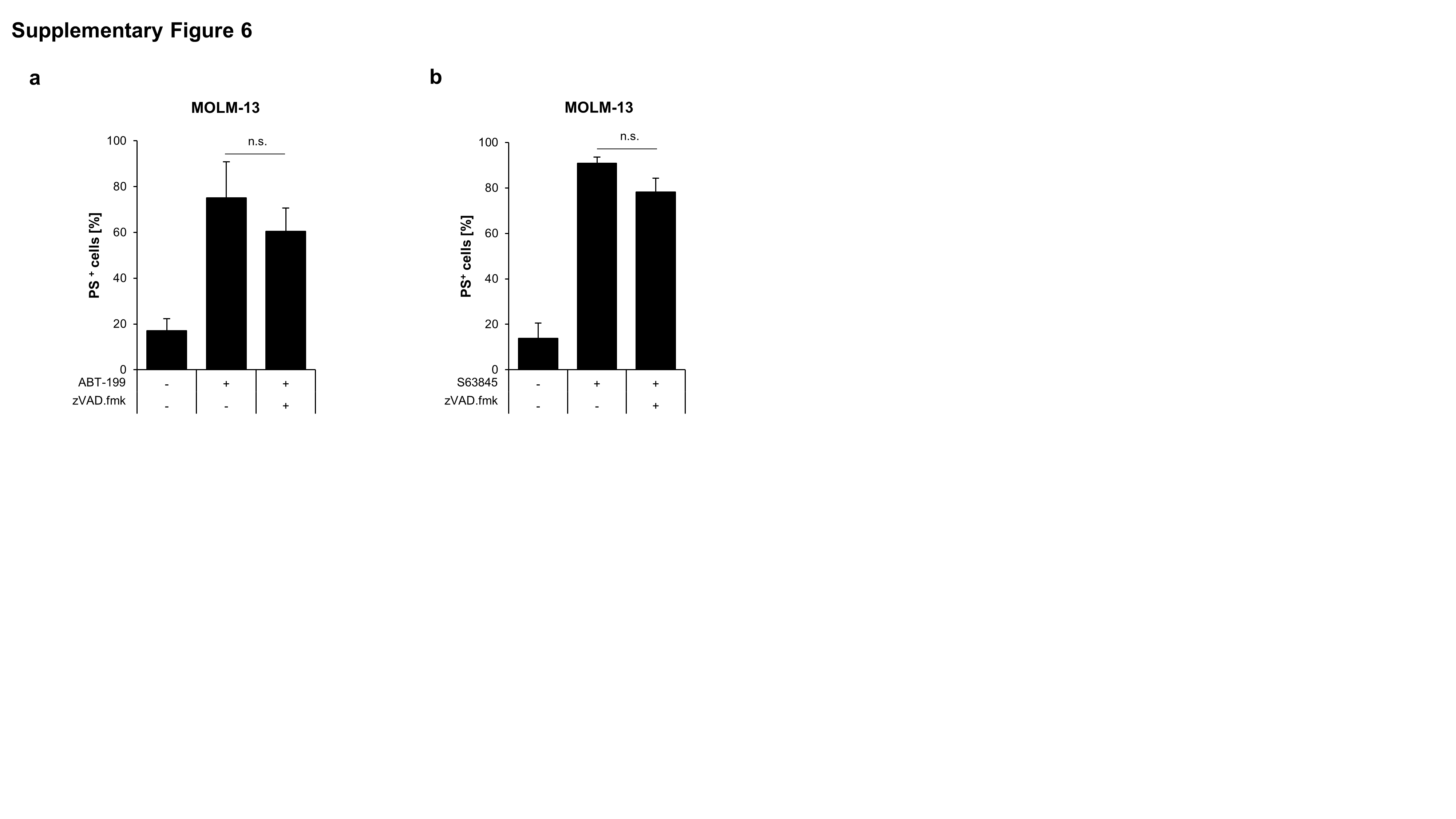

Supplement: Supplementary file 6 — Supplementary Figure 6 [file 41419_2019_2156_MOESM6_ESM.tif]

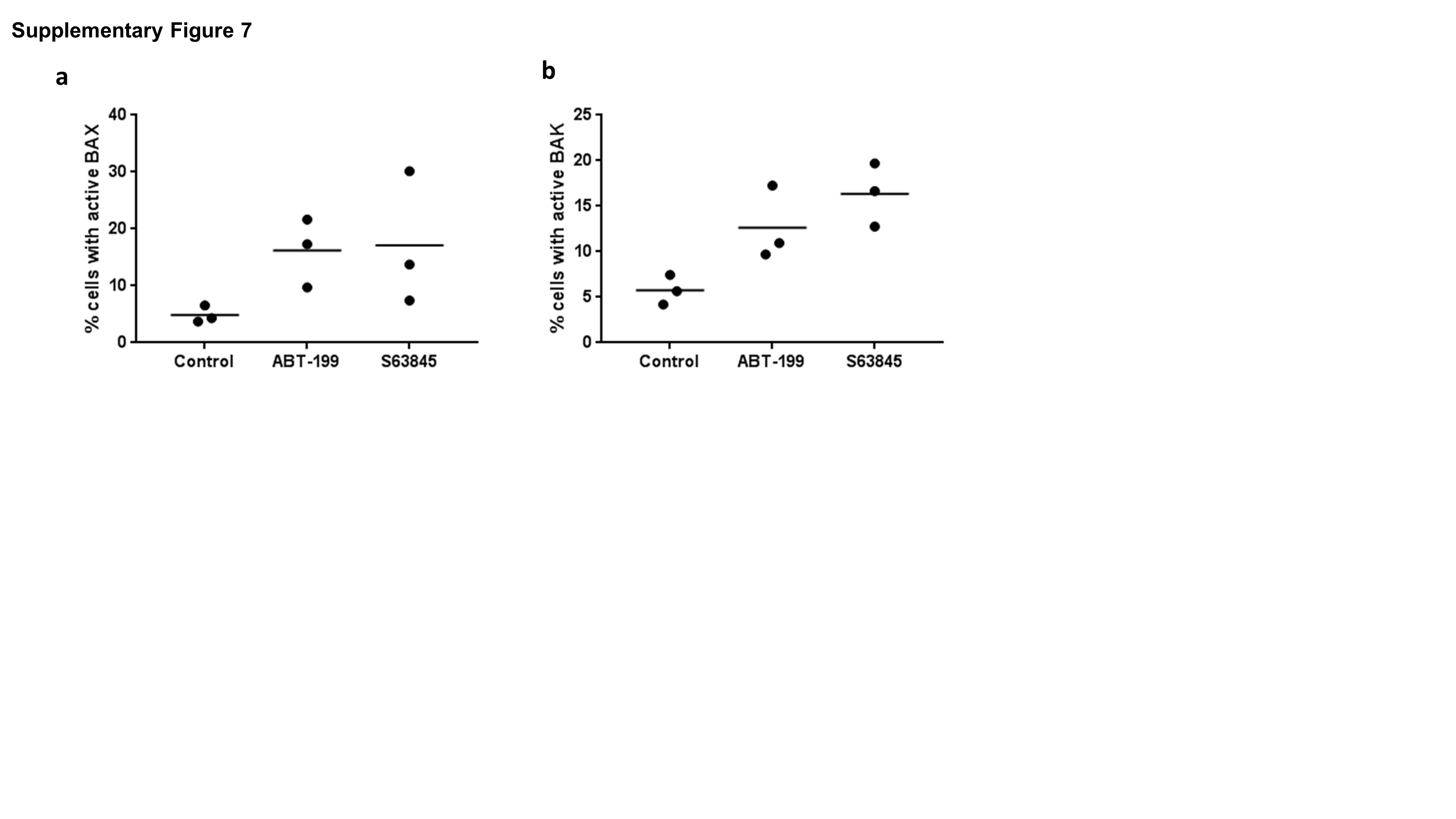

Supplement: Supplementary file 7 — Supplementary Figure 7 [file 41419_2019_2156_MOESM7_ESM.tif]

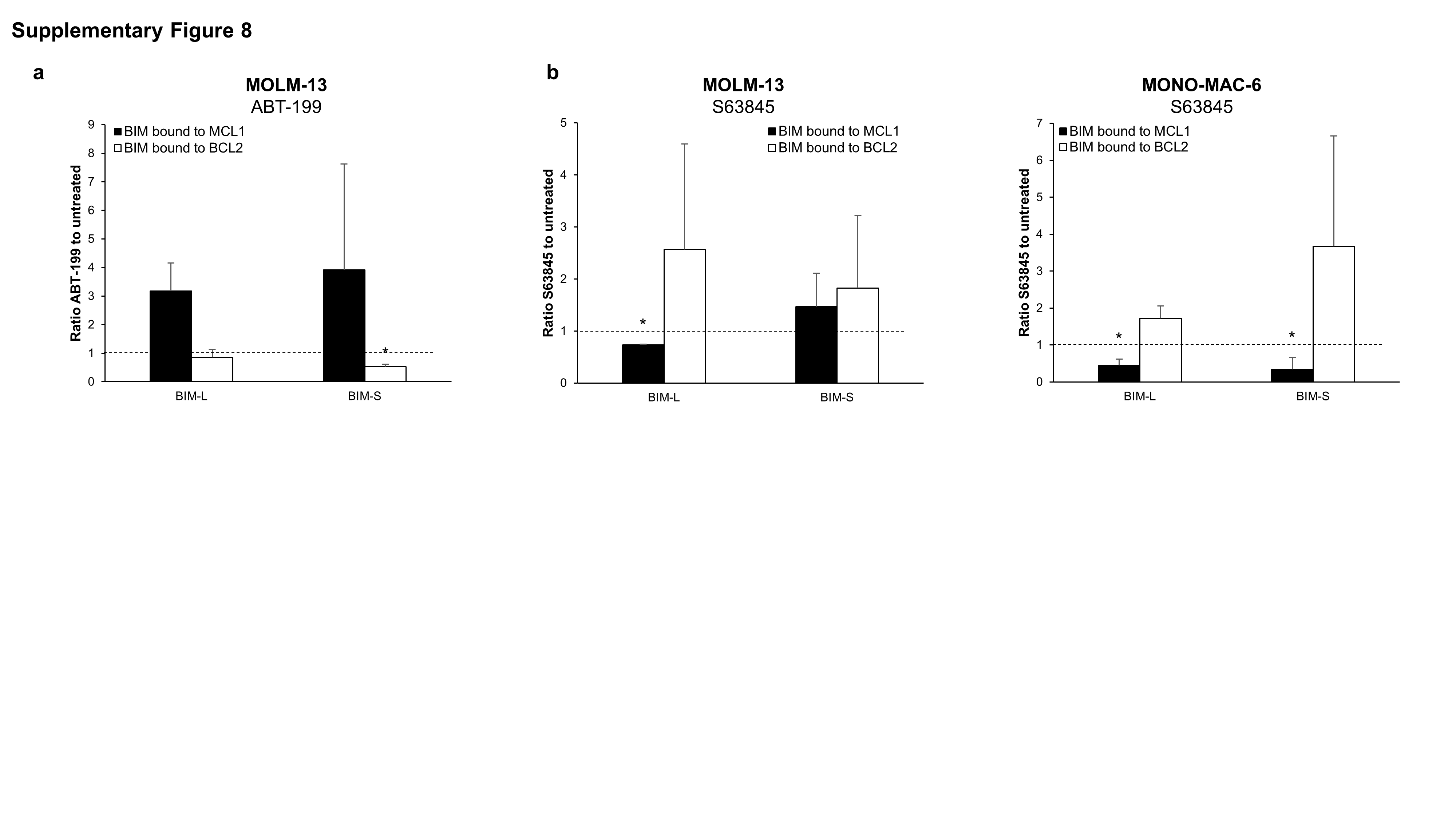

Supplement: Supplementary file 8 — Supplementary Figure 8 [file 41419_2019_2156_MOESM8_ESM.tif]
