# Supplementary material for: Side-by-side comparison of BH3-mimetics identifies MCL-1 as a key therapeutic target in AML
Source: Cell Death Dis. 2019 Dec 4;10(12):917. doi: 10.1038/s41419-019-2156-2 (PMC6892884; doi:10.1038/s41419-019-2156-2)
Supplement: Supplementary file 9 — Supplementary Figure Legends [file 41419_2019_2156_MOESM9_ESM.docx]

**Supplementary Figure Legends**

**Supplementary Fig. 1 Comparison of S63845 and ABT-199**

Primary AML cells (a) or AML cell lines (b) were treated with S63845 or ABT-199 as shown in Figure 1. EC_50_ values (μM) were calculated and plotted. Data shown are mean and individual data points. *, p<0.05; **, p<0.01.

**Supplementary Fig. 2 Effect of BH3-mimetics on non-malignant CD34+ cells**

(a) CD34+ cells were treated with BH3-mimetics for 24 h prior to analysis of apoptosis using Annexin V-FITC staining and flow cytometry. To normalize the differences in spontaneous apoptosis specific apoptosis is displayed as mean and SD (n=4). (b) CD34+ cells were treated with BH3-mimetics before assessing colony formation. Colonies were distinguished into colony-forming unit-granulocyte, erythrocyte, macrophage, megakaryocyte (CFU-GEMM), colony-forming unit-granulocyte, macrophage (CFU-GM) or burst-forming unit-erythroid (BFU-E), normalized to untreated controls (set to 100%) and shown as mean and SD (n=3). (c) Representative images are shown upon treatment with 1 μM of BH3-mimetics.

**Supplementary Fig. 3 BH3-mimetics induce apoptosis in AML cell lines**

AML cell lines were treated with BH3-mimetics for 24 h prior to analysis of apoptosis using Annexin V-FITC staining and flow cytometry. Data shown are mean of three independent experiments performed in triplicate.

**Supplementary Fig. 4 ABT-199 and S63845 synergistically induce cell death**

MOLM-13 (a) or MONO-MAC-6 (b) cells were incubated with different concentrations of ABT-199 and S63845 for 24 h prior to analysis of apoptosis using Annexin V-FITC staining and flow cytometry. Data shown are mean + SD (n=3-4). Tables below the graphs display the calculated combination index (CI) for the tested concentrations, with a CI< 0.8 indicating synergistic drug interactions.

**Supplementary Fig. 5 Sensitivity to BH3-mimetics does not correlate with expression levels of antiapoptotic BCL-2 proteins**

(a) Expression of BCL-2 as assessed by Western blotting was quantified and correlated with the EC_50_ for ABT-199. (b) Expression of BCL-xL as assessed by Western blotting was quantified and correlated with the EC_50_ for A1331852. (c) Expression of MCL-1 or BCL-xL as assessed by Western blotting was quantified and correlated with the EC_50_ for S63845.

**Supplementary Fig. 6 Upon prolonged incubation BH3-mimetics induce caspase-independent cell death**

MOLM-13 cells were incubated with 1 μM ABT-199 (a) or S63845 (b) with or without addition of the caspase inhibitor zVAD.fmk for 24 h prior to analysis of apoptosis using Annexin V-FITC staining and flow cytometry. Data shown are mean + SD (n=3-4). n.s.; not significant.

**Supplementary Fig. 7 ABT-199 and S63845 induce BAX and BAK activation in primary AML cells**

Activation of BAX (a) or BAK (b) was assessed in primary AML cells upon treatment with ABT-199 or S63845 for 24h by intracellular staining with antibodies against conformationally active BAX or BAK and flow cytometry. Data shown are mean and individual data points of 3 primary AML samples.

**Supplementary Fig. 8 Quantification of BIM displacement**

Cells were treated with ABT-199 (a) or S63845 (b) before immunoprecipitation of BCL-2 or MCL-1 as displayed in Figure 5. Quantification of BIM_L_ or BIM_S_ binding to MCL-1 or BCL-2 was done by densitometry. Data shown are mean + SD (n=3-5). For better orientation, the dotted line at y=1 indicates the level of untreated control cells. *, p<0.05.
